# Prexasertib treatment induces homologous recombination deficiency and synergizes with olaparib in triple-negative breast cancer cells

**DOI:** 10.1186/s13058-019-1192-2

**Published:** 2019-09-06

**Authors:** Chinnadurai Mani, Shirisha Jonnalagadda, Jojireddy Lingareddy, Sanjay Awasthi, William H. Gmeiner, Komaraiah Palle

**Affiliations:** 1 Department of Cell Biology and Biochemistry, Texas Tech University Health Sciences Centre, Lubbock, TX 79430 USA; 2 Present Address: Loyola Academic Degree PG College, Old Alwal, Secunderabad, Telangana 500010 India; 3 Department of Internal Medicine, Texas Tech University Health Sciences Centre, Lubbock, TX 79430 USA; 4 Department of Cancer Biology, Wake Forest School of Medicine, Winston-Salem, NC 27157 USA

**Keywords:** Prexasertib, Olaparib, TNBC, RAD51, CHK1 and homologous recombination

## Abstract

**Background:**

Breast cancer remains as one of the most lethal types of cancer in women. Among various subtypes, triple-negative breast cancer (TNBC) is the most aggressive and hard to treat type of breast cancer. Mechanistically, increased DNA repair and cell cycle checkpoint activation remain as the foremost reasons behind TNBC tumor resistance to chemotherapy and disease recurrence.

**Methods:**

We evaluated the mechanism of prexasertib-induced regulation of homologous recombination (HR) proteins using 20S proteasome inhibitors and RT-PCR. HR efficiency and DNA damages were evaluated using Dr-GFP and comet assays. DNA morphology and DNA repair focus studies were analyzed using immunofluorescence. UALCAN portal was used to evaluate the expression of *RAD51* and survival probability based on tumor stage, subtype, and race in breast cancer patients.

**Results:**

Our results show that prexasertib treatment promotes both post-translational and transcriptional mediated regulation of BRCA1 and RAD51 proteins. Additionally, prexasertib-treated TNBC cells revealed over 55% reduction in HR efficiency compared to control cells. Based on these results, we hypothesized that prexasertib treatment induced homologous recombination deficiency (HRD) and thus should synergize with PARP inhibitors (PARPi) in TNBC cells. As predicted, combined treatment of prexasertib and PARPi olaparib increased DNA strand breaks, γH2AX foci, and nuclear disintegration relative to single-agent treatment. Further, the prexasertib and olaparib combination was synergistic in multiple TNBC cell lines, as indicated by combination index (CI) values. Analysis of TCGA data revealed elevated *RAD51* expression in breast tumors compared to normal breast tissues, especially in TNBC subtype. Interestingly, there was a discrepancy in *RAD51* expression in racial groups, with African-American and Asian breast cancer patients showing elevated *RAD51* expression compared to Caucasian breast cancer patients. Consistent with these observations, African-American and Asian TNBC patients show decreased survival.

**Conclusions:**

Based on these data, RAD51 could be a biomarker for aggressive TNBC and for racial disparity in breast cancer. As positive correlation exists between *RAD51* and *CHEK1* expression in breast cancer, the in vitro preclinical data presented here provides additional mechanistic insights for further evaluation of the rational combination of prexasertib and olaparib for improved outcomes and reduced racial disparity in TNBC.

## Background

Drugs that damage DNA and interfere with replication and other DNA-dependent cellular processes have historically been among the most successful for anti-cancer therapy [1, 2] and remain particularly important for treatment of triple-negative breast cancer (TNBC) and other malignancies for which targeted therapies are non-existent or relatively less effective. The utility of DNA damaging treatments is often limited by resistance which can occur via any of several mechanisms including elevated DNA repair [3, 4], increased drug efflux [5, 6], increased drug metabolism [6, 7], epigenetic silencing of drug targets [8, 9], epithelial-mesenchymal transition [10, 11], and cell cycle checkpoint activation [12, 13]. Various inhibitors targeting the above-mentioned resistance pathways have been developed to potentiate DNA damage and overcome chemo-resistance in cancer cells. In specific, inhibitors of cell cycle checkpoint signaling have gained recent popularity because of their potent anti-cancer activity.

Cell cycle check point proteins, checkpoint kinase 1 (CHK1) and checkpoint kinase 2 (CHK2), are regulated by ataxia telangiectasia and rad3-related protein ATR (ATR) and ataxia telangiectasia mutated (ATM), respectively, during DNA damage. However, numerous studies have shown that there is a potential crosstalk between these kinases. CHK1 is found to be activated by ATM in response to radiation [14, 15]. Similarly, CHK2 is also shown to be activated by ATR in response to cisplatin or radiation [16, 17]. Due to their importance in repairing DNA damage induced by chemotherapy, CHK1 inhibitors such as prexasertib, PF-477736, and MK-8776 have shown promising anti-cancer activity in multiple cancer types [18–22]. Among them, the CHK1 inhibitor (CHK1i) prexasertib has demonstrated successful single-agent and combination activity with various chemotherapeutic agents in phase I and phase II clinical trials [23–27]. Mechanistically, prexasertib is an adenosine triphosphate (ATP)-competitive inhibitor of CHK1, which inhibits CHK2 to a lesser extent [28]. Prexasertib treatment in cancer cells induces replication stress and abrogates S-phase cell cycle arrest and increases DNA double-strand breaks (DSB) [28–30], leading to cell death.

In recent years, poly (ADP-ribose) polymerase (PARP) inhibitors (PARPi) have demonstrated clinical utility in the treatment of malignancies with deficiencies in homologous recombination DNA repair (i.e., HRD). Initially, PARPi were approved for treating breast cancer susceptibility protein (BRCA)-deficient breast and ovarian cancer. However, recent studies show that in addition to BRCA deficiency, deficiency in other proteins required for homologous recombination (HR) including RAD51 [31], Fanconi anemia complementation group (FANC)D2 [32], FANCA [33], phosphatase and tensin homolog (PTEN) [34], Ligase-4 [35], partner and localizer of BRCA2 (PALB2) [36], CHK [37] can all sensitize cancer cells to PARPi. Among the various PARPi, olaparib has been approved by the Food and Drug Administration (FDA) for the treatment of ovarian, breast, and prostate cancers while talazoparib has been approved for treating human epidermal growth factor receptor 2 (HER2)-negative locally advanced or metastatic breast cancer or BRCA-deficient breast cancers and rucaparib and niraparib have been approved for treating ovarian cancer.

Here, for the first time, we evaluate a CHK1i (prexasertib) in combination with a PARPi (olaparib) for cytotoxicity and potential synergy in TNBC cell lines. Interestingly, we find that prexasertib affects the stability of the HR proteins BRCA1 and RAD51 in a proteasome-dependent manner and induces HR deficiency (HRD) in TNBC cells. As our data provide additional mechanistic insights for therapeutic synergy of the prexasertib and olaparib combination, analysis of The Cancer Genome Atlas (TCGA) data for gene expression was evaluated and revealed *RAD51* is a poor prognostic marker for TNBC patients. Additionally, *RAD51* expression levels were much higher in African-American and Asian breast cancer patients compared to Caucasians, suggesting RAD51 as a biomarker for racial disparities in breast cancer. We propose PARPi+CHK1i as a novel combination therapy to more effectively treat TNBC with potential to improve outcomes for all TNBC patients and to reduce disparities.

## Methods

### Cell lines, culture method, and reagents

Human TNBC cell lines MDAMB231, MDAMB453, and MDAMB468 were purchased from ATCC, Manassas, VA. All three cell lines were cultured in Dulbecco’s modified Eagle medium (Corning, Manassas, VA), supplemented with 10% fetal bovine serum (Omega Scientific Inc., Tarzana, CA) and 1% penicillin-streptomycin (50 U/mL, 50 μg/mL, Invitrogen, Eugene, OR). Prexasertib (Sellechem, Houston, TX), olaparib (Sellechem, Houston, TX), epoxomicin (Sigma, St. Louis, MO), and MG132 (Sellechem, Houston, TX) were dissolved in DMSO and used at the specified concentrations and times as indicated. The following primary antibodies were used for western blotting: RAD51 (Santa Cruz Biotechnology, Santa Cruz, CA), BRCA1 (Santa Cruz Biotechnology, Santa Cruz, CA), γH2AX (Millipore, Billerica, MA), pCHK1 S296 (Cell Signaling, Danvers, MA), CHK1 (Santa Cruz Biotechnology, Santa Cruz, CA), and GAPDH (Santa Cruz Biotechnology, Santa Cruz, CA).

### HR Dr-GFP assay

A Dr-GFP reporter assay is used to measure HR activity, as previously described [38]. Plasmids were obtained from Addgene (Watertown, MA). In brief, MDAMB231 cells were stably transfected with pDr-GFP and selected for puromycin resistance (5 μg/mL). Upon 60% confluence, these stably transfected cells were transfected with plasmid I-Sce1. Restriction enzyme I-Sce1 cuts the reporter plasmid and initiates the GFP expression when the damage is repaired by HR. GFP-positive cells were measured by flow cytometry using a BD Accuri (BD Biosciences) flow cytometer.

### Protein expression by western blot

As previously described [39], cells were placed on ice and washed twice with ice-cold PBS, and cell lysates were collected using cytoskeletal (CSK) buffer (10 mM PIPES at pH 6.8, 100 mM NaCl, 300 mM sucrose, 3 mM MgCl_2_, 1 mM EGTA, 0.1 mM ATP, 0.1% Triton X-100 freshly supplemented with 1 mM dithiothreitol, 1× protease and phosphatase inhibitors with EDTA). Bradford reagent was used to estimate protein content, and the proteins were equilibrated using CSK buffer with 6× Laemmli buffer and heated at 100 °C for 15 min. The proteins were resolved on gradient polyacrylamide gels and then transferred onto nitrocellulose membrane using Biorad Trans-Blot Turbo system. The membranes were blocked using 2.5% blocking grade blocker (BioRad, USA) in 1× Tris-buffered saline in 0.1% Tween 20 (TBST) and incubated with the primary antibody overnight on a rocking platform at 4 °C. Membranes were than washed three times with 1× TBST, and secondary antibody was added and incubated further for an hour. The membranes were again washed three times with 1× TBST and exposed to Western lightning plus ECL (Perklin Elmer, USA) and developed in a dark room with Konica Minolta equipment.

### Cell cycle analysis

After drug treatment, cells were trypsinized and washed with ice-cold PBS. Cells were then re-suspended in ice-cold ethanol and incubated overnight at − 20 °C. After incubation, cells were washed with PBS, stained with propidium iodide (PI) (Invitrogen, Eugene, OR), and analyzed for cell cycle profile by flow cytometry using a BD Accuri (BD Biosciences) flow cytometer.

### Micronucleus and mitotic catastrophe assay

To observe micronuclei and morphological changes associated with mitotic catastrophe, treated cells were fixed with ice-cold methanol for 5 min, on ice. The cells were washed thrice with PBS and stained with DAPI (Invitrogen, Eugene, OR) for 5 min and imaged using an Axiovision microscope.

### RNA isolation and real-time PCR

Total RNA was extracted from cells at indicated time intervals using a Purelink RNA isolation kit from Ambion (Carlsbad, CA). One microgram of RNA was reverse-transcribed using a high-capacity cDNA reverse transcription kit (Invitrogen, Eugene, OR), as per the manufacturer’s protocol and as previously described [40]. The primers for different genes amplified (*BRCA1*, *RAD51*, and *GAPDH*) were purchased from Bio-Rad. Amplification of PCR products was quantified using SYBR green dye (ABI), and fluorescence was monitored on a QuantStudio 12 K Flex detection system. Melting curve analysis was done for each amplicon. The 2−ΔΔCt method was used for quantitation with *GAPDH* as an endogenous control.

### Immunofluorescence

Cells were seeded into fluorodish (World Precision Instruments) and incubated overnight for adherence. After drug treatment, cells were fixed with 4% formaldehyde for 10 min at room temperature. Cells were then permeabilized using 0.2% Triton X-100 in PBS for 3 min. Cells were washed and blocked using 10% goat serum in PBS for 40 min. After three washes with PBS, cells were incubated overnight at 4 °C with primary antibodies (BRCA1, RAD51, and γH2AX) in PBS, followed by incubating with fluorescent secondary antibodies (Molecular Probes, Eugene, OR) for 2 h at room temperature. Cells were mounted with Vectashield containing DAPI and analyzed for focus formation [38], using a Nikon Eclipse TE confocal microscope.

### Comet assay

Comet assays were performed under alkaline conditions using the CometAssay Kit (Trevigen, Gaithersburg, MD), as per the manufacturer’s instructions and as we previously reported [41]. In brief, cells were treated 24 h with DMSO, 250 nM prexasertib, or 25 μM olaparib (or their combinations); harvested; and combined with 1% low melting point agarose at a ratio of 1:10 (v/v). Slides were immersed in a lysis solution for 30 min and electrophoresed in a horizontal electrophoresis apparatus. The samples were subsequently fixed in 70% ethanol and dried. Slides were then stained with SYBR green to visualize cellular DNA. Fluorescence images were analyzed using the ImageJ comet program to demarcate the “head” and “tail” regions of each comet. The comet tail area was measured, and calculations were averaged from three independent experiments.

### Clonogenic survival assay

Three hundred fifty cells per well were seeded into 6-well culture plates and incubated overnight for adherence. Cells were then treated with DMSO or various concentrations of prexasertib and/or olaparib and cultured for colony formation over a period of 9–12 days. After colony formation, growth medium was removed, cells were washed with ice-cold PBS thrice, and then fixed in ice-cold methanol for 5 min. Methanol was replaced with 1% w/v crystal violet (Invitrogen, Eugene, OR) for staining, and after 10 min, the wells were washed under gentle tap water, and plates were allowed to dry at room temperature. Colonies were then imaged and counted.

### Statistical analysis

Student’s *t* test was performed to estimate statistical significance using GraphPad Prism 8.0 software.

## Results

### Prexasertib downregulates HR proteins in TNBC

**Fig. 1:**
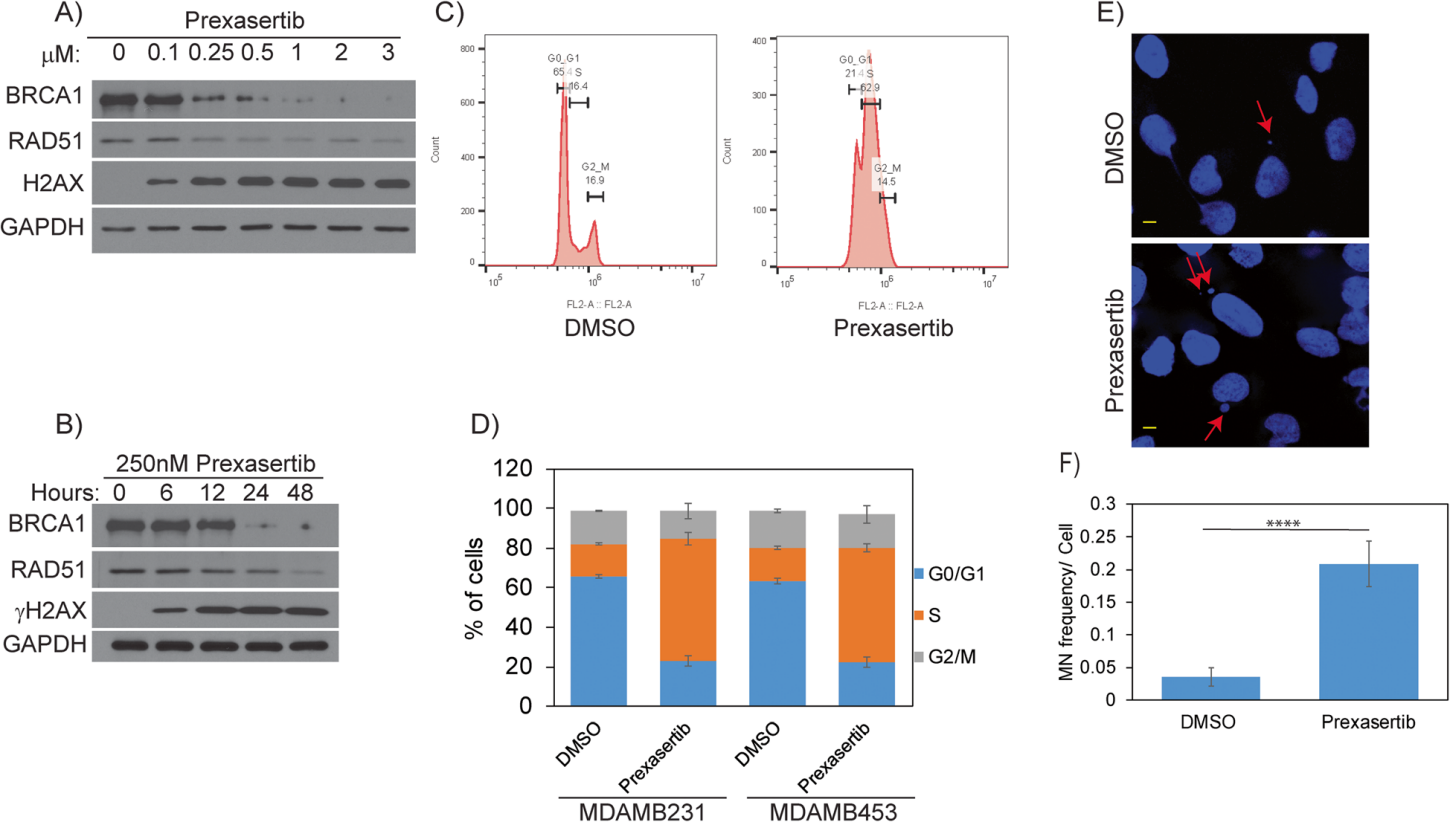
**a** Expression of HR proteins in MDAMB231 cells treated with indicated concentrations of prexasertib for 24 h. **b** Expression of HR proteins in MDAMB231 cells treated with 250 nM prexasertib at time points indicated. **c** Cell cycle profile of MDAMB231 cells treated with 250 nM prexasertib for 24 h. **d** Histogram representation of cell cycle profile in TNBC cells treated with 250 nM prexasertib for 24 h. Error bars represent standard deviation from three independent experiments. **e** Representative images of MN formation in MDAMB231 cells treated with 250 nM prexasertib for 24 h. **f** More than 170 cells from three independent experiments were scored, and MN frequency/cell with standard error is represented in the histogram. Scale bar represents 5 μM (****p < 0.0001)

TNBC is not amenable to targeted therapy, and while cytotoxic chemotherapy is often effective, drug resistance is a serious concern contributing to the high mortality of this malignancy. In our previous studies, we showed that CHK1 inhibition with prexasertib or PF-477736 downregulated expression of RAD51 [42] in colon cancer cells. We sought to determine if TNBC cells could be induced into a state of HR deficiency by CHK1 inhibitor treatment, which would potentially render them susceptible to PARPi. To determine if CHK1 inhibition downregulates RAD51 in TNBC, we treated the TNBC cell line MDAMB231 with different concentrations of prexasertib (Fig. 1a) for 24 h. Consistent with our previous findings, prexasertib decreased RAD51 protein levels in a dose-dependent manner in MDAMB231 cells. As RAD51 is the downstream effector protein of the Fanconi anemia-BRCA pathway (FA-BRCA) which mediates HR, we further analyzed BRCA1 levels in these cells. Interestingly, BRCA1 levels were decreased similar to RAD51 in response to prexasertib treatment. Consistent with previous observations, prexasertib-treated cells exhibited increased γH2AX levels in a dose-dependent manner up to a concentration of 500 nM prexasertib, after which the levels seem to be saturated. Based on these results, we used 250 nM prexasertib to further determine the time at which prexasertib downregulates BRCA1 and RAD51 levels and induces maximum DNA damage response signal by measuring γH2AX. As shown in Fig. 1b, BRCA1 and RAD51 levels diminish starting from 6 h of prexasertib exposure to undetectable levels at 48 h.

It is well known that HR proteins are predominantly expressed during S and G2/M phases of the cell cycle and are lowest or undetected in G0/G1 phase [43]. To rule out the possibility that the observed reduction in BRCA1 and RAD51 levels upon prexasertib treatment is not due to arrest of cells in G0/G1 phase, we performed cell cycle analysis of these cells by flow cytometry. Similar to previously published studies [29, 30], both MDAMB231 and MDAMB453 cells treated with 250 nM prexasertib for 24 h were predominantly arrested in S phase of the cell cycle, ruling out any cell cycle effects contributing to the observed reduced expression of BRCA1 and RAD51 proteins (Fig. 1c, d). Further, pharmacological inhibition of CHK1/2 has been shown to abrogate S and G2 cell cycle arrest, which allows the cancer cells with damaged DNA to enter mitosis and results in the increase of micronucleus (MN) formation [44, 45]. Similarly, prexasertib-treated MDAMB231 cells showed a statistically significant (*p* < 0.0001) increase in the levels of MN formation compared to DMSO-treated control cells (Fig. 1e, f).

### Prexasertib promotes ubiquitin proteasome-mediated degradation of BRCA1 and RAD51

**Fig. 2:**
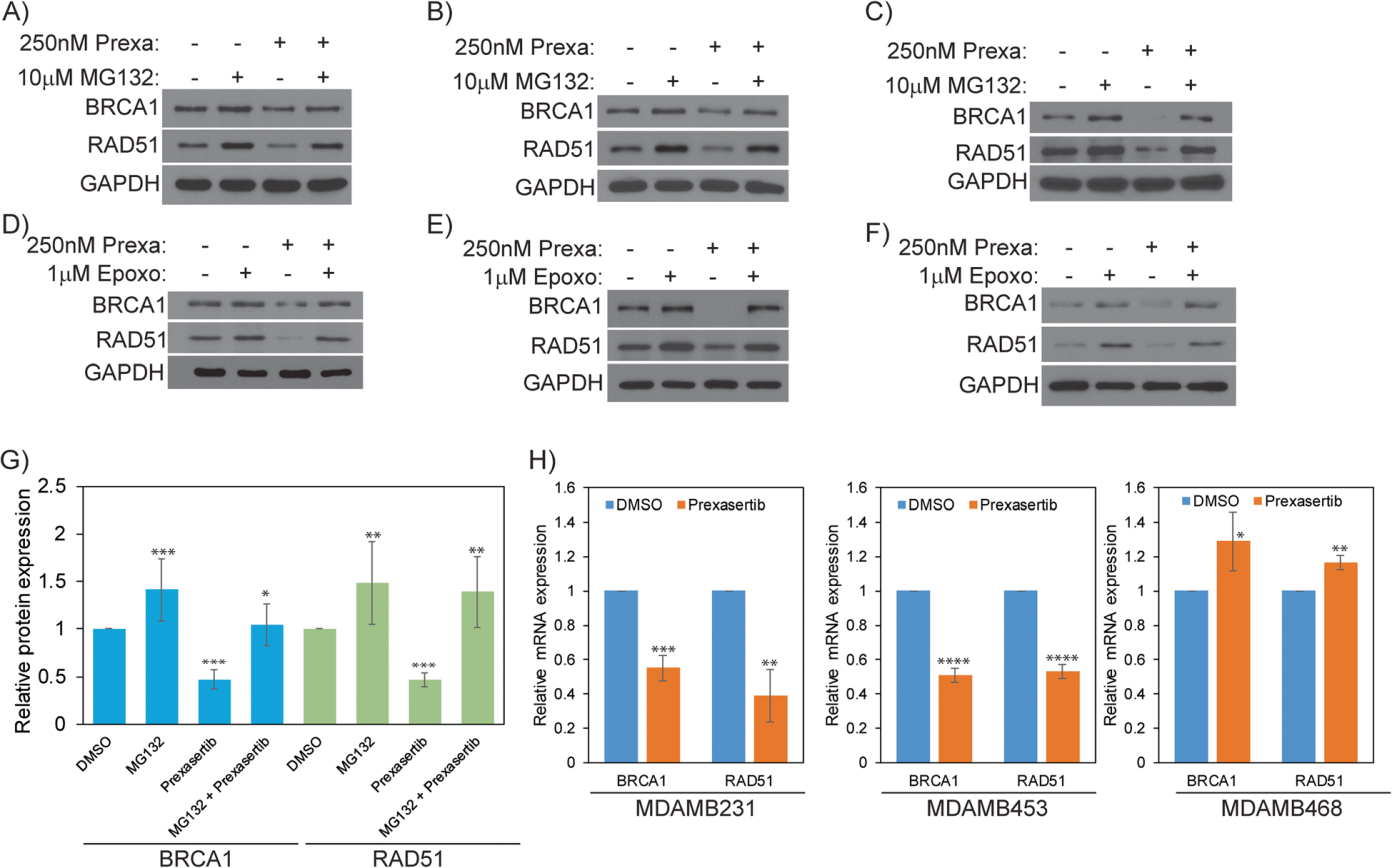
**a** MDAMB231, **b** MDAMB453, and **c** MDAMB468 cells were treated with 250 nM prexasertib for 24 h and co-treated with or without 10 μM MG132 for the last 16 h. Protein expression was evaluated by western blot. **d** MDAMB231, **e** MDAMB453, and **f** MDAMB468 cells were treated with 250 nM prexasertib and co-treated with or without 1 μM epoxomicin for 24 h with protein expression analyzed by western blot. **g** Densitometry analysis for BRCA1 and RAD51 expression in all three TNBC cell lines are represented in histogram with standard deviation as error bars. **h** RT-qPCR analysis of prexasertib-treated TNBC cells compared with vehicle (DMSO). Fold difference with standard deviation is represented as histogram (***p < 0.001)

To investigate the mechanism for BRCA1 and RAD51 downregulation in prexasertib-treated cells, we used two proteasomal inhibitors (MG132 and epoxomicin) and also evaluated their transcript levels by qRT-PCR. First, we exposed MDAMB231 cells to prexasertib for 24 h and for the last 16 h, and these cells were co-treated with ± MG132, an inhibitor of the 20S proteasome. Remarkably, the diminished BRCA1 and RAD51 levels in prexasertib-treated MDAMB231 cells were reverted close to the levels of DMSO-treated cells when co-treated with MG132 (Fig. 2a). To check whether the observed results are cell line specific, we repeated similar studies in two additional TNBC cell lines MDAMB453 and MDAMB468. Consistently, prexasertib treatment downregulated expression of BRCA1 and RAD51 in TNBC cells, and MG132 co-treatment was protective from degradation (Fig. 2b, c).

To rule out that the observed results are limited to MG132, we used another proteasomal inhibitor epoxomicin, which also inhibits the 20S proteasome. Similar to MG132, cells co-treated with epoxomicin also inhibited prexasertib-induced downregulation of BRCA1 and RAD51 in all three TNBC cell lines evaluated (Fig. 2d–f). Additionally, densitometry analysis of BRCA1 and RAD51 proteins from multiple experimental blots confirmed that proteasomal inhibition protects BRCA1 and RAD51 from degradation in prexasertib-treated cells (Fig. 2g).

To further evaluate transcript levels of BRCA and RAD51, each of the TNBC cell lines were treated with either DMSO or prexasertib for 24 h, and mRNA levels were analyzed relative to *GAPDH* by qRT-PCR. As shown in Fig. 2h, transcript levels of *BRCA1* and *RAD51* were downregulated in prexasertib-treated MDAMB231 and MDAMB453 cells. However, in MDAMB468 cells, we did not observe downregulation of these genes. In fact, we noticed a mild increase in the expressions of *BRCA1* and *RAD51* in these cells*.* Together, these results indicate that prexasertib-mediated downregulation of BRCA1 and RAD51 could be primarily due to proteasome-mediated degradation; however, downregulation at the transcript levels was also observed in some, but not all, TNBC cell lines. Nevertheless, further studies are required to address cell line-dependent variations and contribution of genetic and epigenetic factors to such variations.

### Prexasertib induces HR deficiency and sensitizes TNBC cell to olaparib-induced DNA damage

**Fig. 3:**
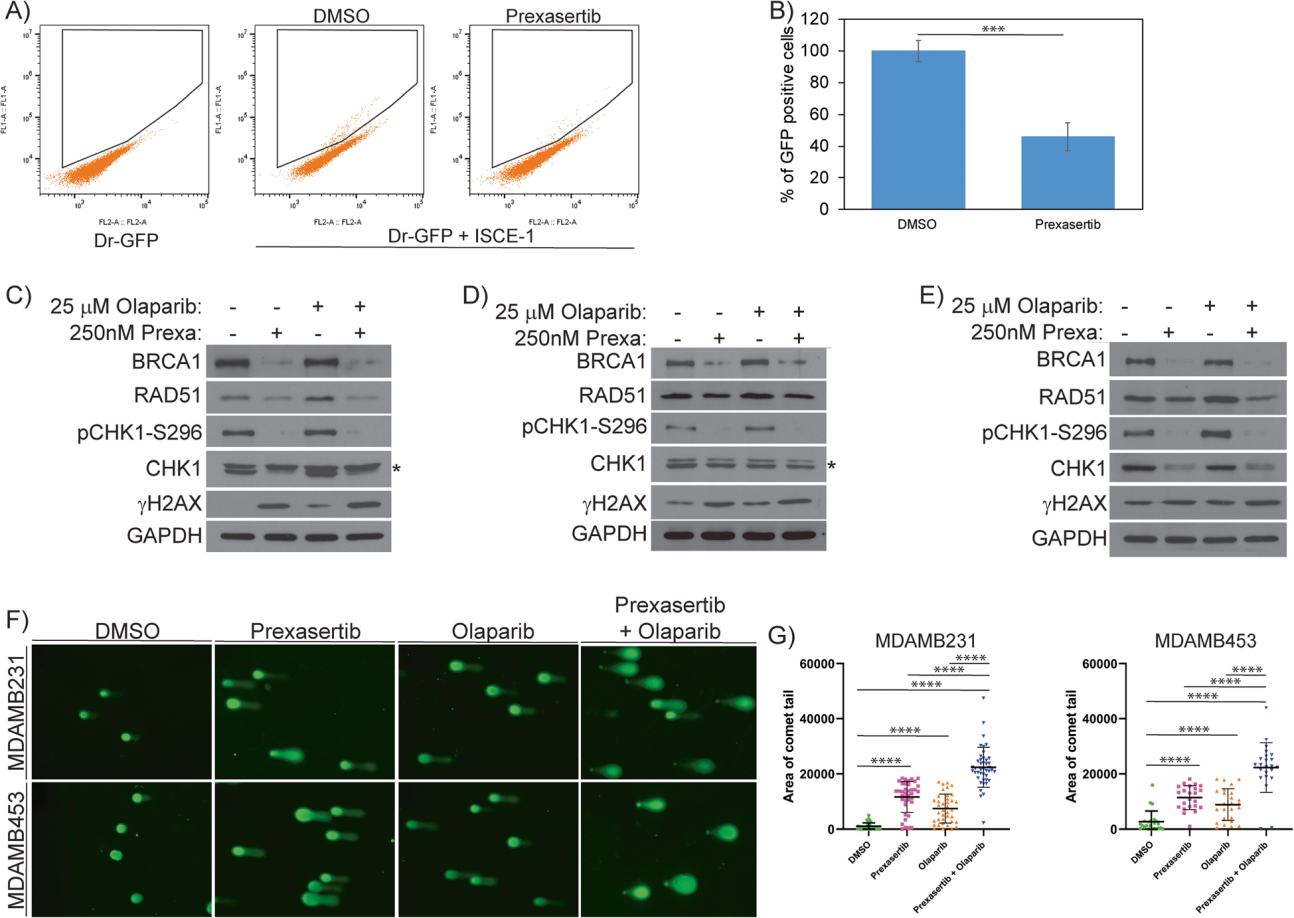
**a** MDAMB231 cells were transfected with Dr-GFP and selected using 5 μg/ml puromycin. Stably expressing cells were transfected with ISce-1 and analyzed for GFP^+^ cells using flow cytometry 48 h after transfection. Twenty-four hours before analysis, cells were treated with DMSO or 250 nM prexasertib. **b** Histogram representation of GFP^+^ cells from three independent experiments with standard deviation as error bars. **c** MDAMB231, **d** MDAMB453, and **e** MDAMB468 cells were treated with 250 nM prexasertib and/or 25 μM olaparib for 24 h and analyzed for protein expression using western blot. **f** Comet assay representative images in MDAMB231 and MDAMB453 cells treated with DMSO or 250 nM prexasertib or 25 μM olaparib or their combination for 24 h. **g** Analysis of comet tail area in more than 25 cells from three different experiments with their standard deviation as the error bars (***p < 0.001 and ****p < 0.0001)

The FA-BRCA tumor suppressor pathway proteins BRCA1 and RAD51 are important for normal replication fork progression and maintenance of DNA integrity at collapsed forks, which is mediated by HR [46–49]. Thus, cells deficient in components of the FA-BRCA pathway are defective in HR-mediated repair. To assess HR efficiency in prexasertib-treated cells, we adapted the Dr-GFP reporter system-based assay [50, 51]. We generated Dr-GFP-expressing MDAMB231 cells and transfected them with an I-Sce1 expression plasmid to induce DSB in the presence or absence of prexasertib treatment. Cells were analyzed for GFP expression by flow cytometry (Fig. 3a). Consistent with the downregulation of BRCA1 and RAD51, more than 55% reduction in HR efficiency (*p* < 0.001) was observed in prexasertib-treated cells compared to the control cells (Fig. 3b).

Cells with HRD display increased DNA lesions and synthetic lethality upon treatment with PARPi [52]. As our studies show prexasertib treatment induces HRD, we evaluated the FDA-approved PARPi olaparib, in combination with prexasertib. Three TNBC cell lines were treated with prexasertib, olaparib, or the combination and were assessed for the DNA damage response. Consistently, in cells treated with prexasertib, levels of pCHK1-S296, BRCA1, and RAD51 were downregulated, yet γH2AX levels were increased, consistent with CHK1 inhibition leading to fork collapse and DSB. Similarly, olaparib activated the DNA damage response as evidenced by CHK1 phosphorylation (pCHK1-S296), increased γH2AX, and increased RAD51 levels in all three TNBC cell lines (Fig. 3c–e). Interestingly, for the olaparib + prexasertib combination, olaparib-induced CHK1 activation was detected (pCHK1-S296) and BRCA1 and RAD51 levels were diminished to undetectable levels. In this combination, we also observe a small increase in γH2AX. To further investigate the extent of DNA lesions upon drug treatment, we evaluated DNA strand breaks in MDAMB231 and MDAMB453 cells using an alkaline comet assay. As shown in Fig. 3f and g, cells treated with the combination of prexasertib and olaparib displayed statistically significant increases (*p* < 0.0001) in comet tail compared to control cells and the individual treatments. These results suggest that prexasertib inhibits CHK1-mediated checkpoint responses and downregulates HR proteins (e.g., BRCA1 and RAD51) attenuating repair efficiency of TNBC cells to olaparib-induced DNA lesions and potentiates DNA damage in these cells.

**Fig. 4:**
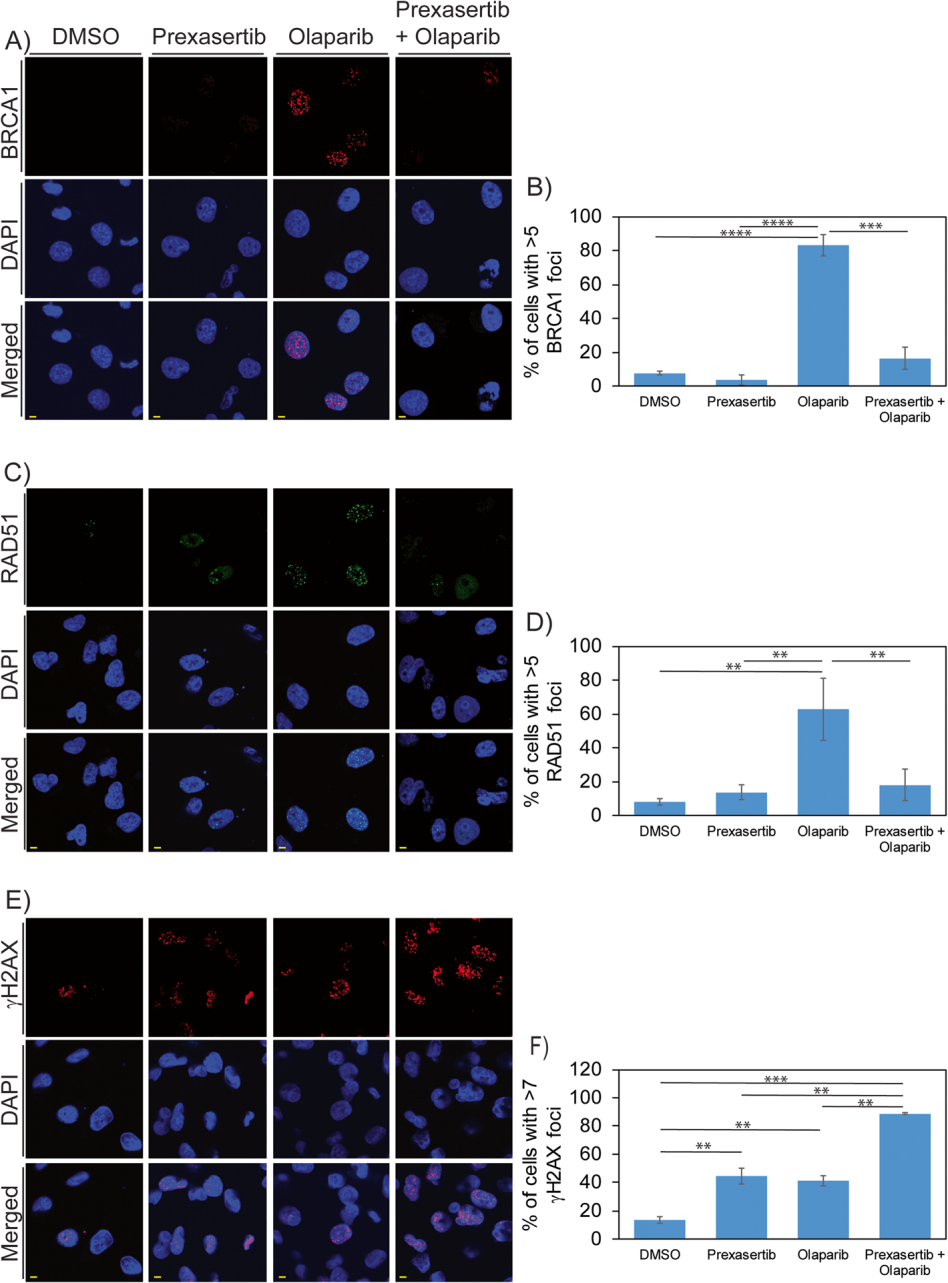
**a** MDAMB231 cells treated with DMSO or 250 nM prexasertib and/or 25 μM olaparib for 24 h were analyzed for BRCA1 foci using immunofluorescence. **b** More than 75 cells from three independent experiments were analyzed for percentage of cells that shows > 5 BRCA1 foci and represented as histogram with standard error. **c** MDAMB231 cells treated with DMSO or 250 nM prexasertib and/or 25 μM olaparib for 24 h were analyzed for RAD51 foci using immunofluorescence. **d** More than 75 cells from three independent experiments were analyzed for percentage of cells that shows > 5 RAD51 foci and represented as histogram with standard error. **e** MDAMB231 cells treated with DMSO or 250 nM prexasertib and/or 25 μM olaparib for 24 h were analyzed for γH2AX foci using immunofluorescence. **f** More than 75 cells from three independent experiments were analyzed for percentage of cells that shows > 7 γH2AX foci and represented as histogram with standard error. Scale bar represents 5 μM (**p < 0.01, ***p < 0.001, and ****p < 0.0001)

To further investigate the effects of HR downregulation on DNA repair in TNBC cells treated with the prexasertib + olaparib combination, we assessed BRCA1 and RAD51 foci and γH2AX foci by immunofluorescence in MDAMB231 cells. BRCA1, RAD51, and γH2AX foci were not detected in most vehicle-treated cells (Fig. 4a–f). Consistent with the western blots (Fig. 3), olaparib treatment significantly increased the number of cells with BRCA1 foci (*p* < 0.0001), RAD51 foci (*p* < 0.01), and γH2AX foci (*p* < 0.01) (Fig. 4a–f). However, in combination treatment, prexasertib attenuates olaparib-induced BRCA1 and RAD51 foci formation (*p* < 0.0001 and *p* < 0.001 respectively) (Fig. 4a–d). Consistent with our comet assay results, γH2AX focus cells were increased to several magnitudes (*p* < 0.01) in cells treated with the combination compared to control and individual drugs (Fig. 4e, f).

### Prexasertib abrogates S-G2 phase checkpoint and induces mitotic catastrophe

**Fig. 5:**
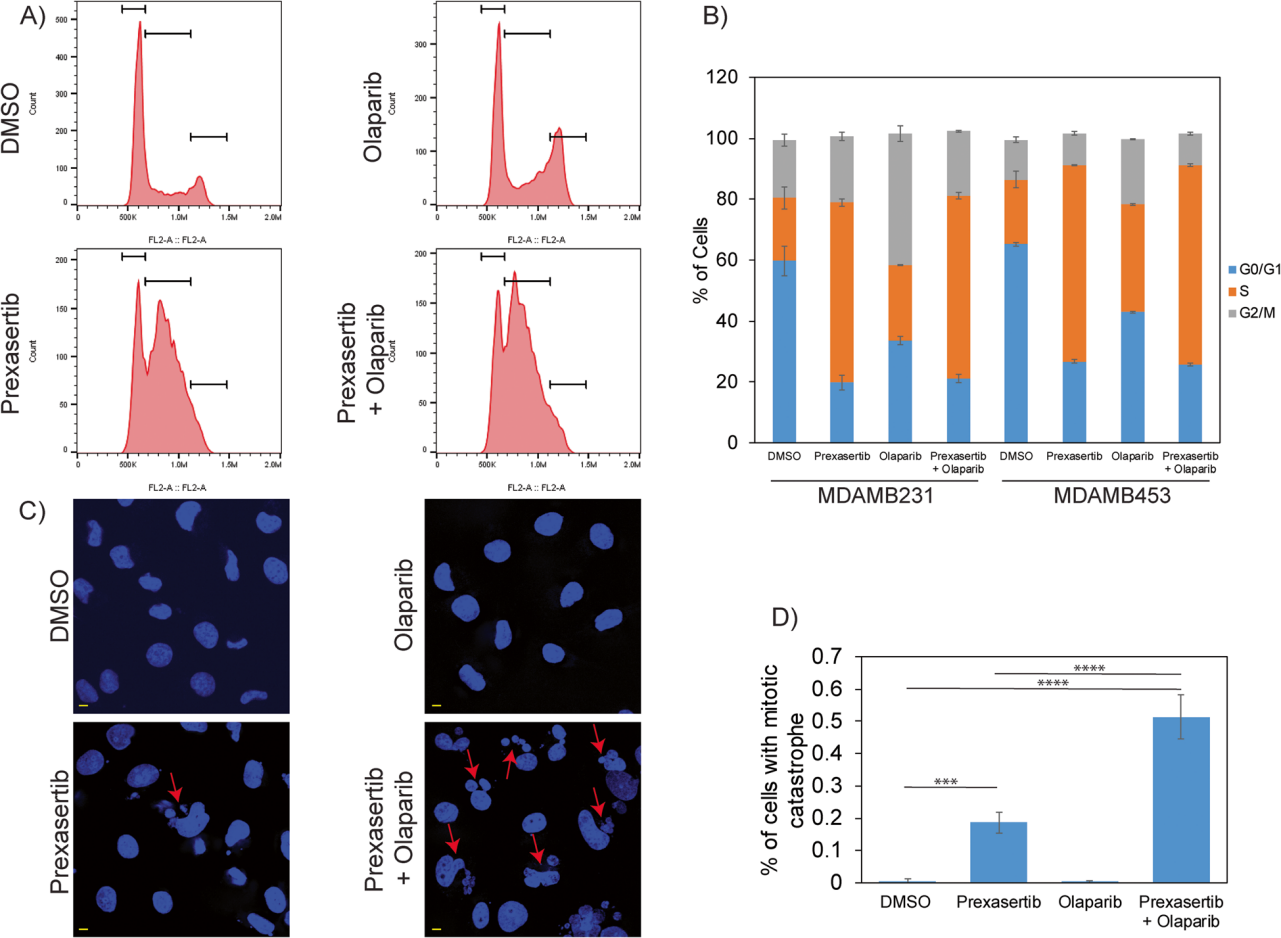
**a** Cell cycle profile of MDAMB231 cells treated with 250 nM prexasertib and/or 25 μM olaparib for 24 h. **b** Histogram representation of cell cycle profile in TNBC cells treated with 250 nM prexasertib and/or 25 μM olaparib for 24 h. **c** Mitotic catastrophe representative images in DAPI-stained nucleus of MDAMB231 cells treated with DMSO or 250 nM prexasertib or 25 μM olaparib or their combination for 24 h. **d** Percentage of cells with mitotic catastrophe analyzed from more than 110 cells from three different experiments is represented with standard error. Scale bar represents 5 μM (***p < 0.001 and ****p < 0.0001)

To gain further insight into the effects of CHK1i+PARPi combination to TNBC cells, we evaluated the cell cycle profile of MDAMB231 and MDAMB453 cells treated with prexasertib + olaparib relative to the individual treatments (Fig. 5a). Prexasertib treatment induces S-phase arrest (Fig. 1c, d), while olaparib mono-treatment increases the percent cells in both S and G2/M phases of the cell cycle. However, the cell cycle profile for the combination shows a perturbed G2/M phase arrest (Fig. 5a, b). This observation may be due to prexasertib overriding G2 arrest in olaparib-treated cells causing cells to undergo mitosis with unrepaired DNA damage, resulting in mitotic catastrophe, which is characterized by a loss of nuclear membrane integrity and fragmented morphology [53]. We evaluated the structure and morphology of MDAMB231 cell nuclei to determine if drug treatment induced mitotic catastrophe. While prexasertib treatment alone increased mitotic catastrophe compared to control, the prexasertib + olaparib combination increased mitotic catastrophe by threefold (Fig. 5c, d). We did not observe significant levels of such morphologic changes in olaparib-treated cells.

### Synergy of the prexasertib + olaparib combination in TNBC cells

**Fig. 6:**
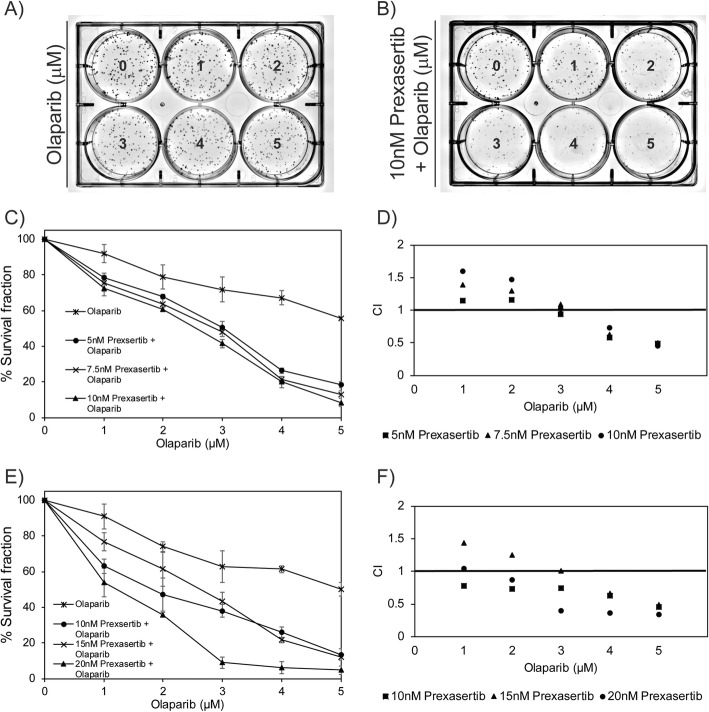
**a**, **b** Colony assay plates of MDAMB231 cells with various concentration of prexasertib and/or olaparib. **c** Survival fraction of MDAMB231 cells treated with various concentrations of prexasertib and/or olaparib. **d** Combination index values of MDAMB231 cells treated with various concentrations of prexasertib and/or olaparib. **e** Survival fraction of MDAMB453 cells treated with various concentrations of prexasertib and/or olaparib. **f** Combination index values of MDAMB453 cells treated with various concentrations of prexasertib and/or olaparib

To evaluate the prexasertib + olaparib combination for synergy in TNBC cells, we performed clonogenic assays in MDAMB231 and MDAMB453 cells (Fig. 6a, b). We selected three prexasertib concentrations and five olaparib concentrations that were less than the IC-50 values for the individual drugs and evaluated the effects of the combined drugs at each of these concentrations relative to the single drugs using a colony formation assay. Results in MDAMB231 cells showed decreased colony formation for the combination relative to olaparib mono-treatment (Fig. 6c). To quantify potential synergistic interaction for the prexasertib + olaparib combination, we used COMPUSYN to analyze combinational index (CI) values. Out of the 15 combinations evaluated, 7 were synergistic and 2 were additive (Fig. 6d). Similarly, in MDAMB453 cells, reduced colony formation was detected for the combination relative to single-agent olaparib (Fig. 6e) and 11 synergistic and 2 additive effects were observed for the 15 combinations evaluated in these cells (Fig. 6f).

### Clinical relevance of *RAD51* expression in breast cancer patients

**Fig. 7:**
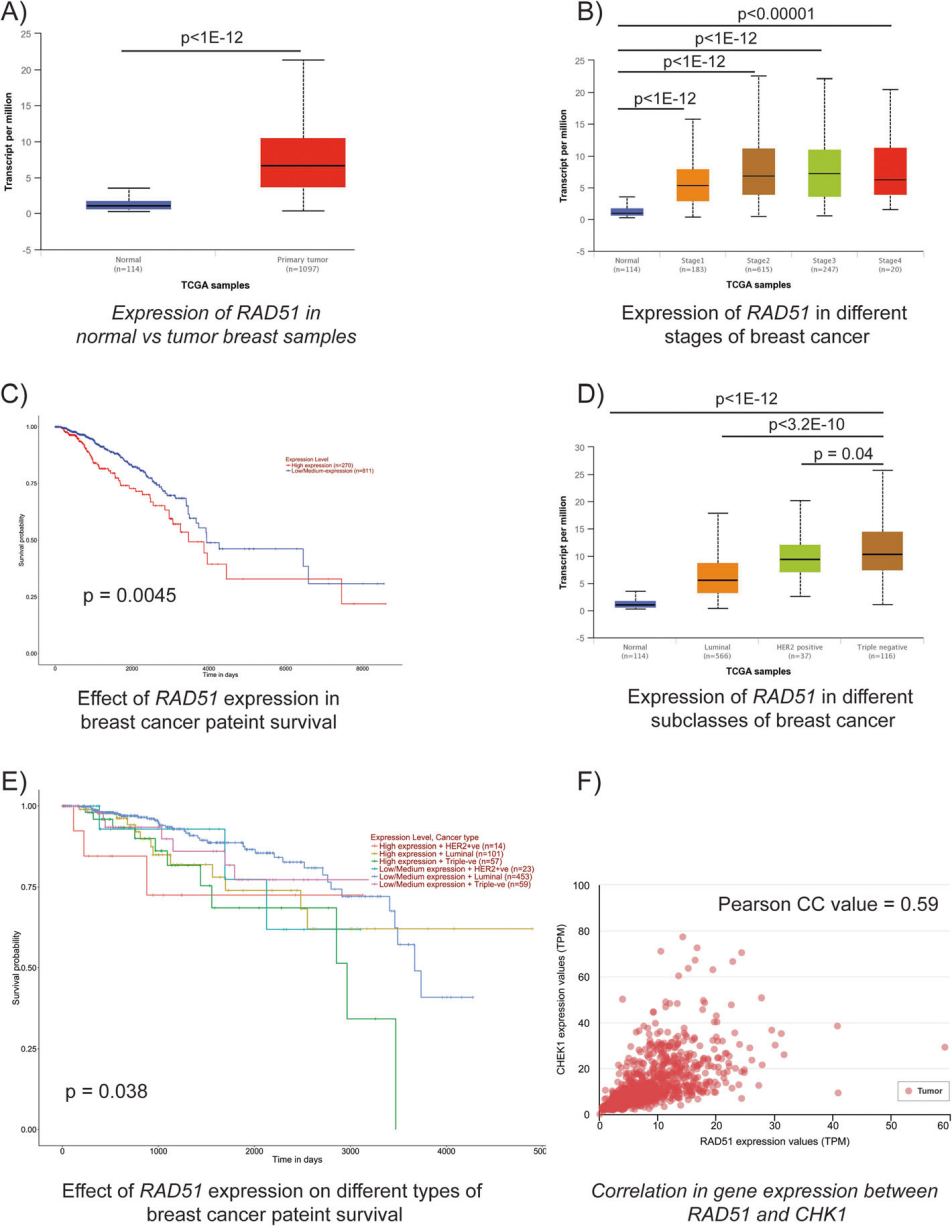
UALCAN portal analysis of breast cancer samples from the TCGA database. **a** Comparison of *RAD51* expression between normal and tumor breast cancer samples. **b** Expression of *RAD51* in different stages of breast cancer. **c** Survival probability between breast cancer patients with high and low/medium *RAD51* expression. **d** Expression of *RAD51* in different subtypes of breast cancer. **e** Survival probability between breast cancer patients with different subtypes and *RAD51* expression. **f** Correlation of *RAD51* and *CHEK1* gene expression in breast cancer

Since RAD51 is the downstream effector repair protein in FA-BRCA pathway-mediated HR, we evaluated its expression in breast cancer patients included in the TCGA database using the UALCAN portal [54]. Interestingly, *RAD51* is overexpressed in primary breast tumors (*n* = 1097) compared to normal tissues (*n* = 114) (Fig. 7a). We also observed an increase in *RAD51* expression in stage 1 thru 4 compared to normal samples (Fig. 7b). Consistent with these observations, breast cancer patients with overexpressed *RAD51* (*n* = 270) displayed decreased survival probability (*p* = 0.0045) compared to the patients with low/medium-level *RAD51* expression (*n* = 811) (Fig. 7c).

Among the various subtypes of breast cancer, TNBC is the most aggressive and lacks options for targeted therapy [55]. Remarkably, our analysis displays that TNBC tumors express higher *RAD51* compared to either the Her2^+^ or luminal subtypes (Fig. 7d). Further, TNBC patients with elevated *RAD51* expression have reduced survival probability compared to other subtypes regardless of *RAD51* expression (Fig. 7e). Furthermore, we also evaluated whether there is any positive correlation between *RAD51* and *CHEK1* expressions in breast cancer. Remarkably, we observed a positive correlation between *RAD51* and *CHEK1* expressions with a Pearson CC value of 0.59, a border value between moderate and strong correlation (Fig. 7e).

**Fig. 8:**
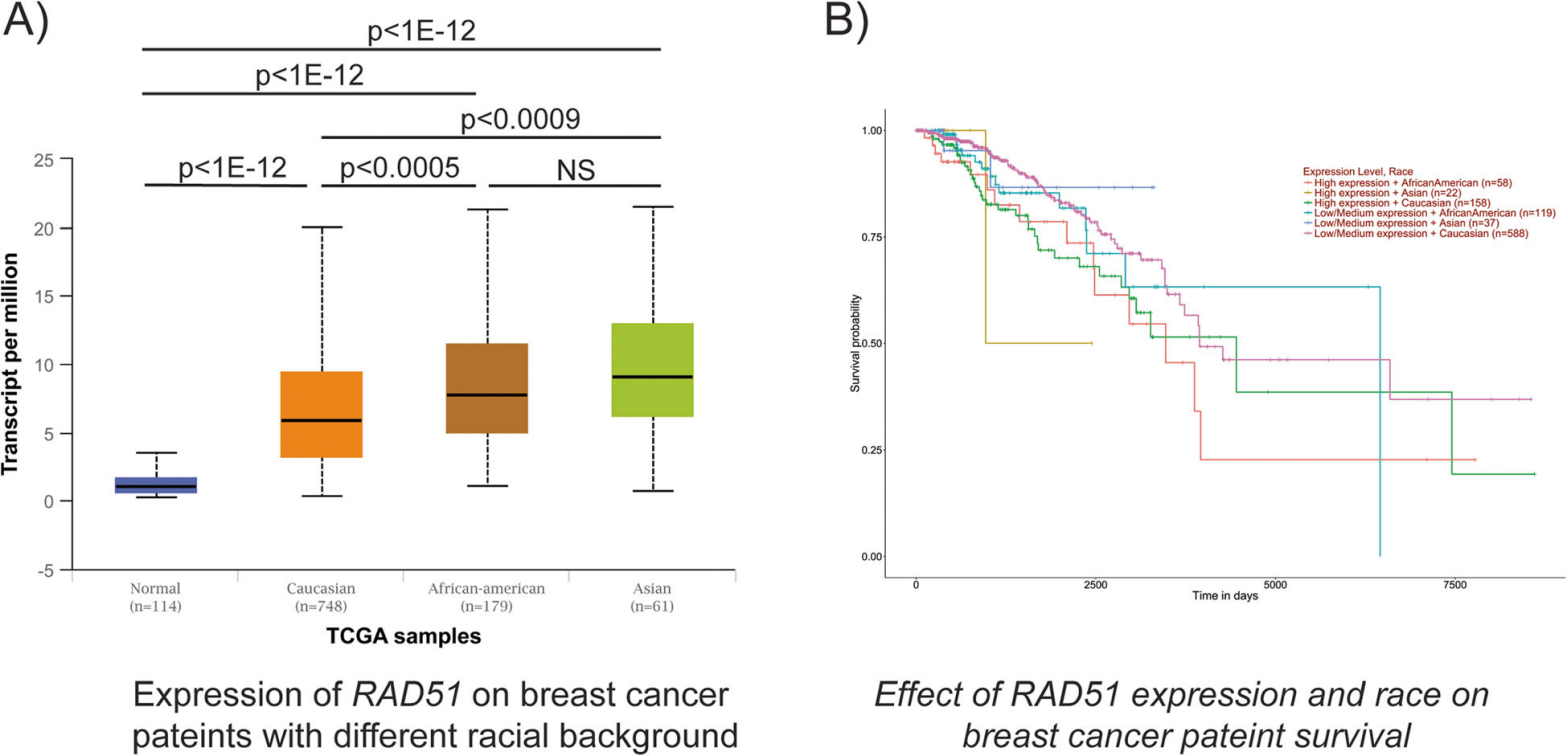
**a** *RAD51* expression in patients with different racial background. **b** Survival probability of patients with different race and *RAD51* expression

Since epidemiological and other population-based studies indicate existence of racial disparities in different ethnic populations for breast cancer in breast cancer progression and therapy [56], we further analyzed the expression of *RAD51* between different races and their survival probability. Both African-Americans (*n* = 179) and Asians (*n* = 61) display relatively elevated *RAD51* expression compared to Caucasians (*n* = 748) (Fig. 8a). Further, both African-American and Asian breast cancer patients with high *RAD51* levels display reduced survival probability (Fig. 8b) consistent with RAD51 levels contributing to racial disparities. These observations together with our preclinical data showing the CHK1i prexasertib downregulates Rad51 and decreases HR repair and sensitizes TNBC cells to the PARPi olaparib indicate the prexasertib + olaparib could effectively treat TNBC patients and reduce racial disparities.

## Discussion

Overall, our results show that pharmacological inhibition of CHK1 by prexasertib downregulates BRCA1 and RAD51 levels, predominantly by affecting protein stability. Inhibition of proteasomal degradation with two different 20S proteasome inhibitors (MG132 and epoxomicin) gave a consistent inhibition of prexasertib-induced downregulation of both BRCA1 and RAD51 in all three TNBC cell lines evaluated (Fig. 2a–f). Interestingly, RT-qPCR shows differential regulation among the three TNBC cell lines. Specifically, MDAMB231 and MDAMB453 cells display decreased *BRCA1* and *RAD51* upon prexasertib treatment; however, these genes were upregulated upon prexasertib treatment in MDAMB468 cells. CHK1 is known to interact and phosphorylate RAD51 on threonine 309 region, which is important for RAD51 nuclear focus formation after exposure to hydroxyurea [57]. To our knowledge, we did not come through studies that show CHK1 induced phosphorylation of BRCA1; however, CHK2-induced phosphorylation of BRCA1 at serine 988 site has been previously reported [58]. As mentioned previously, prexasertib has the ability to knock down CHK2 to an extent in addition to CHK1. Collectively, these results confirm that protein stability is the predominant mechanism by which prexasertib is regulating BRCA1 and RAD51 in TNBC cells but indicate transcriptional response to drug treatment occur and may be important in some contexts.

The decreased RAD51 and BRCA1 levels in response to prexasertib treatment of TNBC are associated with reduced foci for these proteins following DNA damage and with increased sensitivity to DNA damaging agents. Prexasertib treatment downregulates RAD51 expression, but does not completely knock out RAD51 levels (Fig. 3c). Therefore, the residual RAD51 present in prexasertib-treated cells may contribute to focus formation, as prexasertib by itself induces DNA damage (Fig. 4c, d). However, this increase is not statistically significant compared to the control cells and does not effectively perform HR repair as shown in Fig. 3a. Our results contrast with previous studies which showed siRNA knockdown of CHK1 affected only the focus-forming ability of RAD51 but did not show changes in RAD51 or BRCA protein stability in response to prexasertib treatment. Capitalizing on the HRD observed with prexasertib treatment (Fig. 3a), we hypothesized that the olaparib + prexasertib combination would be synergistic to BRCA-proficient TNBC cells. In line with our hypothesis, both comet assay and mitotic catastrophe assay show enhanced DNA damage in the prexasertib and olaparib combination compared to the individual treatments. Specifically, we observed only 0.2% of olaparib-treated TNBC cells displayed characteristics of mitotic catastrophe (Fig. 5d), a result consistent with most of olaparib-treated cells undergoing S or G2/M arrest enabling sufficient time to repair DNA damage via BRCA1- and RAD51-mediated HR. However, the combination of prexasertib and olaparib showed a substantial increase of nearly threefold in the percent of cells that undergo mitotic catastrophe (Fig. 5c, d). These data show convincingly that CHK1 inhibition by prexasertib allows olaparib-treated TNBC cells to enter mitosis without repairing damage at either the S or G2/M checkpoint, ultimately resulting in mitotic catastrophe, genomic instability, and cell death.

We recently reported that the CHK1 inhibitors PF-477736 and prexasertib attenuated F10-induced RAD51 activation in colorectal cancer cells [42]. Others reported the gemcitabine + olaparib combination induced RAD51 focus formation in pancreatic cancer cells which was attenuated upon co-treatment with CHK1 inhibitors (MK-8776 or prexasertib) [30, 59]. Prexasertib showed effective anti-tumor activity against preclinical tumor models when combined with doxorubicin, cyclophosphamide, irinotecan, and cisplatin [60]. Additionally, it also showed efficacy in acute lymphoblastic leukemia models in combination with imatinib, dasatinib, and clofarabine [29].

However, the mechanisms behind these observations were not studied. To our knowledge, the present study is the first to investigate in further detail the mechanism of BRCA1 and RAD51 regulation by prexasertib showing it is primarily at the levels of protein stability but also with effects via transcriptional regulation in some TNBC cells.

It is interesting to note that *RAD51* is highly expressed in the TNBC subtype compared to other breast cancer subtypes (Fig. 7). In addition, TNBC patients with high *RAD51* expression displayed reduced survival. Further, African-Americans and Asians have high *RAD51* expression and less survival probability (Fig. 8a, b). Based on our results, prexasertib alone or in combination with olaparib may be effective in treating patients based on the cancer subtype, race, and ethnicity and which improve outcomes.

## Conclusion

Recently, several clinical trials with prexasertib as monotherapy or in combination with other chemotherapeutic agents have been initiated [61]. Relevant to the current study, a phase I trial has been initiated to study the prexasertib + olaparib combination in patients with metastatic or unresectable solid tumors [62]. The present studies provide novel mechanistic insight into the therapeutic potential of this combination, and it will be of interest to determine if this combination displays promising activity in cancer patients. Our work has potential implications for improved response to PARPi in malignancies other than ovarian and prostate, where malignancy-dependent HRD causes synthetic lethality.

## Data Availability

All data generated or analyzed during this study are included in this research article and its supplementary information files.

## Abbreviations

TNBC: Triple-negative breast cancer
HR: Homologous recombination
HRD: Homologous recombination deficiency
CHK1: Checkpoint kinase 1
CHK2: Checkpoint kinase 2
PARP: Poly (ADP-ribose) polymerase
BRCA: Breast cancer susceptibility protein
FANC: Fanconi anemia complementation group
PTEN: Phosphatase and tensin homolog
PALB2: Partner and localizer of BRCA2
TCGA: The Cancer Genome Atlas
PARPi: PARP inhibitor(s)
CI: Combination index
ATM: Ataxia telangiectasia mutated
ATR: ATM- and RAD3-related
CHK1i: CHK1 inhibitor(s)
ATP: Adenosine triphosphate
DSB: Double-strand breaks
FDA: Food and Drug Administration
HER2: Human epidermal growth factor receptor 2
DMSO: Dimethyl sulfoxide
GFP: Green fluorescent protein
CSK: Cytoskeletal
TBST: Tris-buffered saline in 20% Tween 20
PBS: Phosphate-buffered saline
PI: Propidium iodide
MN: Micronucleus
DAPI: 4′,6-Diamidino-2-phenylindole
